# Do Insulin Replacement and Omega3 Protect the Male Reproductive Function of the Streptozotocin-Induced Diabetic Mice?

**DOI:** 10.1155/2017/6102985

**Published:** 2017-07-10

**Authors:** Atefe Yaghoubi, Abbas Shahedi, Hakime Akbari, Seyed Noureddin Nematollahi-Mahani

**Affiliations:** ^1^Department of Anatomy, Afzalipour School of Medicine, Kerman University of Medical Sciences, Kerman, Iran; ^2^Department of Anatomy, Faculty of Medicine, Shahid Sadoughi University of Medical Sciences, Yazd, Iran; ^3^Physiology Research Center, Institute of Neuropharmacology, Kerman University of Medical Sciences, Kerman, Iran; ^4^Afzal Research Institute (NGO), Kerman, Iran

## Abstract

Diabetes mellitus (DM), the most common metabolic disease, might affect different organs such as male reproductive system. Experiments have shown that n-3 fatty acids could improve male reproductive function. Present study was performed to examine the effects of omega3 on sperms and testicular parameters in diabetic mice. Adult NMRI male mice were randomly divided into intact and diabetic groups (*n* = 8). Streptozotocin-induced diabetic animals were divided into 4 groups of diabetic-saline (Dia-Sa), diabetic-insulin (Dia-Ins), diabetic-omega3 (Dia-omg3), and diabetic-insulin-omega3 (Dia-Ins-omg3). Following confirmation of diabetes, different treatments including 3 U/100 g insulin subcutaneously and 400 mg/kg omega3 orally were administered, where applicable according to the treatment groups. Thirty-five days later, the sperm number, motility, progression, and normal morphology were determined. Also, testes diameters and structure including germinal epithelium thickness, seminiferous tubule diameters, Leydig cell number, and testosterone level were assessed. Sperm number, viability, fast motility, testes volume, and serum testosterone level decreased insignificantly in the Dia-Sa group compared with the intact animals. Neither insulin replacement nor omega3 administration could significantly improve the outcome. We might conclude that short periods of diabetes could not significantly affect the male reproductive function. In addition, insulin replacement and/or omega-3 supplementation does not have any profound effects on male reproductive system.

## 1. Introduction

One of the most distinguished common health threats in the modern societies is diabetes mellitus (DM). In 2013, the number of diabetics was 3.82 millions in the world. By 2035, it has been estimated that the number of people who suffer from diabetes would reach 5.92 million [1].

DM might cause problems in various body tissues such as retinopathy, nephropathy, and neuropathy. Some studies have suggested the male reproductive system as one of the target tissues. The effects of DM on the male reproductive system include impairment of spermatogenesis and change in the serum testosterone level and seminal fluid volume [2]. However, opposed to the most of the animal studies, some of the human studies indicate that DM has no remarkable effect on the male reproductive function [3].

To withstand deleterious effects of long term exposure of male reproductive systems to DM, antioxidants including ascorbic acid and vitamin E and vitamin D have been proposed [4–6]. In addition, several experiments have revealed the benefits of n-3 fatty acids on male reproduction capacity [7]. Animals need plenty of n-3 fatty acids in their diets due to the absence of suitable fatty acids desaturase enzymes that they cannot form in their body [8]. Animal sperms benefit from long-chain polyunsaturated fatty acids in plasma membrane and other membrane-bond organelles. Linolenic, eicosapentaenoic, and docosahexaenoic acids are members of n-3 fatty acids formulated as the first double bond at the third carbon position from the terminal methyl group [9, 10]. According to the previous studies, n-3 fatty acids can improve sperm motility and reduce morphological abnormalities [11]. However, the impact of a regimen consisting of higher doses of omega3, as the ringleader of n-3 fatty acids, on diabetic male reproductive systems needs to be investigated [12]. Thus, we aimed at this experimental study to investigate the effects of omega3 upon diabetic animal spermatogenesis. In fact, we try to show whether or not omega3 administration may improve male reproductive function in diabetic animals. To assess this goal, we used streptozotocin (STZ), a potential source of oxidative stress, which is commonly used for the induction of DM in animal experimental models including rat and mouse, and evaluated sperm and testis parameters in diabetic and nondiabetic animals following omega3 administration.

## 2. Materials and Methods

### 2.1. Animals and Groupings

All of the experiments were carried out following a permission issued by ethical board committee at Kerman University of Medical Sciences, Kerman, Iran. In this experimental study, 40 adult NMRI male mice (10–12 weeks old, 25–30 g body weight) were randomly allocated into different groups. Mice were maintained in a temperature controlled area with 12 h light/dark period and free access to drinking water and rodents chew. Animals were divided into the intact group and diabetic groups (*n* = 8).

The intact group received no intervention through the experiments (35 days), while the diabetic groups received a single dose of 150 mg Kg^−1^ STZ intraperitoneally [13]. Seventy-two h later the animals were investigated for DM. Animals with a fasting serum glucose level of ≥200 mg/dl were considered as diabetic [14]. The diabetic animals were randomly allocated into four subgroups: diabetic-saline (Dia-Sa) subgroup received 400 *μ*l Kg^−1^ normal saline orally each day by gavages; diabetic-insulin (Dia-Ins) subgroup received daily subcutaneous injection of 3 IU 100 g^−1^ body weight insulin [15]; diabetic-omega3 (Dia-omg3) subgroup received 400 mg Kg^−1^ omega3 [16] (Dr Zahravi Company, Iran)/day orally; and diabetic-insulin-omega3 (Dia-Ins-omg3) subgroup received daily subcutaneous injection of 3 IU 100 g^−1^ body weight insulin and 400 mg Kg^−1^ omega3/day, orally.

After 35 days, duration of spermatogenesis in mice, the mice were anesthetized by chloroform. The right testes and vas deferens with cauda epididymis were removed for evaluation of sperm and testicular parameters.

### 2.2. Assessment of Serum Testosterone Level

After the mice were anesthetized, an incision was created in their chests. About 0.5–1 ml of blood was aspirated from the left ventricle and centrifuged at 300*g* for 20 min. Serum was carefully aspirated by a fine Pasteur pipette into a clean centrifuge tube, sealed, and kept at −20°C until the time of hormone measurement [17]. Serum testosterone level was measured using an ELISA kit (IBL Company, Japan), as recommended by the manufacture.

### 2.3. Assessment of Sperm Parameters

#### 2.3.1. Sperm Motility

After the mice were anesthetized, an incision was created in the inferior part of the abdomen and right vas deferens with cauda epididymis was removed and transferred into 2.5 ml prewarmed Ham's F10 medium (Sigma Company, St. Louis, MO, USA), supplemented with 8 mg/ml bovine serum albumin (Sigma Company, St. Louis, MO, USA). Spermatozoa were squeezed out by cutting the vas deferens. After 30 min incubation at 37°C and 5% CO2 in the humidified air, the sperm motility was evaluated under a light microscope (Nikon TS100, Tokyo, Japan) by using a 400x magnification. At least 200 spermatozoa were analyzed for each specimen (*n* = 8). Motility was reported as the percentage of fast, slow, and immotile sperm per total sperms.

#### 2.3.2. Serm Count

The sperm cells were fixed by formalin/saline and counted using an improved Neubauer chamber. Sperms were counted in 4 large squares, the mean was multiplied by 10^4^, and the result was reported as the number of sperms in milliliter.

### 2.4. Assessment of Morphology and Viability

For evaluation of sperm viability, eosin-nigrosin staining was used. The sperm cells with dark red heads were dead and the cells with the shiny colorless heads were alive. The same slides were used for the morphology assessment. Any sperm with the head, midpiece, and tail anomaly was considered abnormal [18]. These experiments were carried out by two expert examiners blinded to the design of the study, as described elsewhere [19].

### 2.5. Testicular Parameters

After removing the right testis, its volume and dimensions including length, width, and thickness were measured by using a digital balance and a standard digital caliper. The testes were then fixed in 10% formaldehyde and embedded in paraffin. Five *μ*m sections were stained with hematoxylin-eosin. The Leydig cells were counted in 10 randomly selected fields, using an optical light microscope at 400x magnification.

Diameter of seminiferous tubules and the thickness of germinal epithelium were measured in 3 seminiferous tubules selected from 10 microscopic fields at 400x. The thickness of germinal epithelium and the diameter of seminiferous tubules were measured by a light microscope and with the aim of Analysis® software.

### 2.6. Statistical Analysis

Data were expressed as Mean ± SD. Analysis was carried out using one-way analysis of variance (ANOVA) followed by Tukey's post hoc test for comparison of data between the groups. SPSS software version 21 for Windows was used to analyze the data.

## 3. Results

Streptozotocin-induced diabetic animals showed a higher level of fasting blood glucose (>200 mg/dl) when compared with the serum glucose level in the intact animals. Diabetic animals in the different groups were treated carefully due to their progressive weakness which could potentially harm the animals. All of the diabetic animals showed different signs of diabetes, including polydipsia, overeating, polyurea, and weight loss. However, nearly all of the animals could withstand diabetic conditions during the experimental period (35 days).

### 3.1. Serum Testosterone Level and Leydig Cell Number

The blood testosterone level in all the STZ-induced diabetic mice decreased compared with the intact mice but did not reach a significant level. Compared to the Dia-Ins group (1.26 ± 0.30), testosterone nonsignificantly increased in Dia-Ins-omg3 group (1.44 ± 0.73). But the number of Leydig cells was nonsignificantly lower in Dia-Ins-omg3 group in comparison with the Dia-Ins group. For details please refer to Table 1.

### 3.2. Sperm Parameters

**Figure 1:**
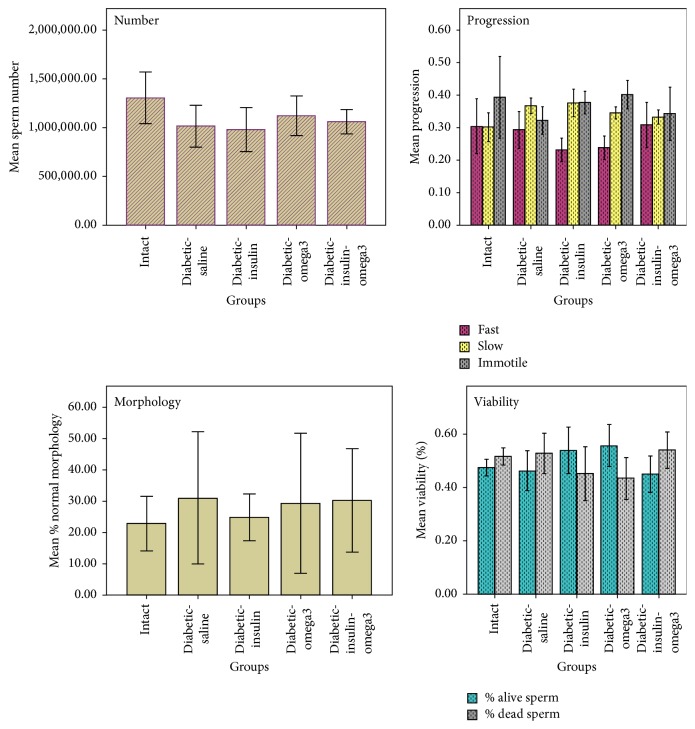
Sperm parameters including number, viability, progression, and normal morphology were evaluated in the intact, diabetic-saline (Dia-Sa), diabetic-insulin (Dia-Ins), diabetic-omega3 (Dia-omg3), and diabetic-insulin-omega3 (Dia-Ins-omg3) groups. There was no statistically significant difference among the groups.

Analysis of sperm parameters showed that sperm number in the intact group was nonsignificantly higher than the diabetic groups. The proportion of the viable sperms was comparable among the groups. However, it was nonsignificantly higher in Dia-omg3 group compared with the other groups (*p* > 0.05). Furthermore, the proportion of sperms with the fast motility in the Dia-Ins-omg3 animals was higher than the other groups, but it did not reach a significant level. Comparison of slow motility in Dia-Sa and Dia-Ins groups showed no significant difference. Sperms with normal morphology were not also significantly different among the groups (Figure 1).

### 3.3. Testicular Parameters

Measurement of the testes dimensions and volume showed no significant difference among the groups (Table 2).

**Figure 2:**
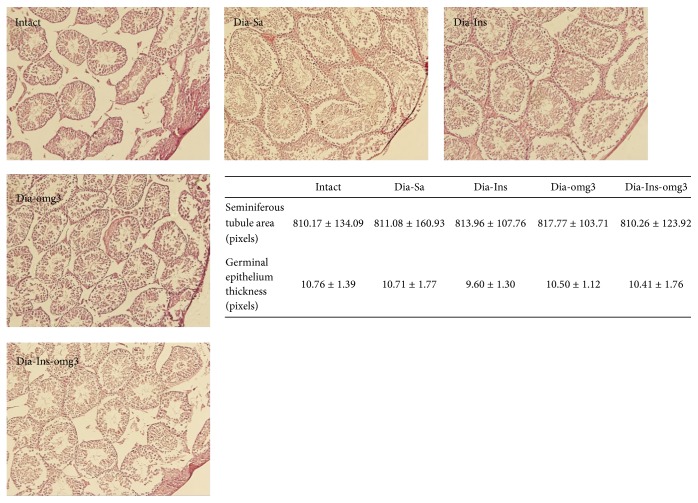
It shows testes structure in the intact, diabetic-saline (Dia-Sa), diabetic-insulin (Dia-Ins), diabetic-omega3 (Dia-omg3), and diabetic-insulin-omega3 (Dia-Ins-omg3) groups. Measurement of seminiferous tubular area and germinal epithelium thickness revealed no statistically significant differences among the groups.

The testes structure appeared nearly similar in diabetic and nondiabetic animals. Neither insulin replacement nor omega3 administration resulted in a significant change in the parameters. Quantitative analysis of the testicular section by Analysis software in the STZ-induced diabetic mice revealed that the thickness of germinal epithelium and the area of seminiferous tubules remained nearly identical in the different groups (Figure 2).

## 4. Discussion

In the present study, we assessed the sperm quality, testicular parameters, and serum testosterone level in STZ-induced diabetic mice after insulin replacement and omega3 supplementation. We administered a regular dose of insulin, which has been approved for the maintenance of glucose in diabetic animals [15] and a daily oral dose of omega3 for 35 days, following onset of diabetes. By this arrangement, we aimed to find out whether these treatments may alter male reproductive function. Sperm number, viability, fast motility, testes volume, and serum testosterone nonsignificantly decreased in diabetic mice that received saline compared with the intact animals.

Mangoli et al. evaluated sperm parameters and chromatin/DNA integrity of spermatozoa in the diabetic mice. Most of the sperm parameters had significantly decreased when compared with the intact animals. However, chromatin integrity remained unchanged among the groups [14]. In addition, Ballester et al. have shown that, in STZ-induced diabetic rats, serum FSH, LH, and testosterone levels significantly reduced in comparison with the control animals [20]. Nikravesh et al. have found that the diameter of seminiferous tubules increased in diabetic rats compared with the control group while Atlay et al., in contrast, reported a significant decrease in the diameter of seminiferous tubules of diabetic animals [21].

In contrast to the most of the animal studies, nearly all of the human studies have revealed no/if any significant differences in the semen samples parameters collected from diabetic men and healthy subjects. Agbaje et al. examined semen from 27 diabetic and 29 nondiabetic men. They found no significant differences in sperm concentration, total sperm count, morphology, and motility of spermatozoa between diabetic and healthy subjects [3].

Also, Petroianu et al. did not find any differences in the sperm motility and seminal concentration between diabetic and nondiabetic men [22]. In addition, Padrón et al., by analyzing semen samples from healthy and diabetic subjects (range 17–22 years), did not report any differences in sperm parameters and serum testosterone level between healthy and diabetic men [23].

In the present study, analysis of sperm parameters and testes structure, in insulin-treated diabetic mice compared with the saline-treated diabetic mice, demonstrated some nonsignificant changes in the reproductive system of diabetic mice. In agreement with our results, Xu et al. found that insulin replacement in diabetic rats inhibited reduction of testosterone level but did not affect other parameters [24].

In our study, we started various treatments in different groups just after the confirmation of DM via an elevated fasting serum glucose level in STZ-induced DM animals and continued it for 35 days, except for the few animals that it was terminated after 32 days due to the sever weakness of animals. By referring to the literature, longer and shorter durations for the induction of reproductive organs alterations can be found, between 12 days and 70 days [3]. No investigation has so far addressed the appropriate time needed for diabetes to affect the male reproductive organs. Whether the prolongation of the diabetic condition might have a serious impact on the reproductive system of male mice has not been reported elsewhere; neither could it be withdrawn from our study. Other influencing conditions, such as the strain of animals (Harbison CE, 2016; Reifsnider PC, 2000; Chitaley K, 2008), age of the animals, and pre- or postdiabetes interventions, should be kept in mind when designing such investigations. Great difference between the animal and human reports that indicate the impact of diabetes on the semen and sperm parameters has raised a debate that a diabetic environment or STZ by itself is responsible for the reproductive changes reported after the induction of diabetes by STZ. In other words, an increase in blood glucose is responsible for reproductive system dysfunction or STZ cytotoxicity on male reproductive organs. Xu et al. (2014), in a recent study, investigated the effects of STZ on male rat's testosterone, sperm, and testis parameters and compared it with STZ-induced rats receiving insulin replacement therapy. They showed a significant decrease in testosterone level compared with the intact rats and a return to the normal value following insulin replacement. They concluded that steroidogenic dysfunction might be a direct or indirect consequence of insulin deficiency that is corrected by insulin replacement, but spermatogenic dysfunction is due to direct effect of STZ on Sertoli cells. They also concluded that STZ-induced diabetic model at early stages (4 weeks) is not appropriate to study diabetic-related spermatogenic changes. Compared with rat model of DM we used a threefold amount of STZ for the induction of DM in mice, and the level of testosterone nonsignificantly decreased below the level of testosterone in the intact mice. However, mean number of Leydig cells, as the main source of testosterone production, was lower in the intact group than diabetic groups. With our data, we cannot conclude that an increase in the number of Leydig cells in the DM mice is proportional to the decrease in testosterone level.

Omega3 has been used in some experiments to improve the fertility rate in domestic animals and rodents [25, 26]. Omega3 might improve sperm motility and progression through sperm flagellum movement enhancement. Here, we used omega3 orally for the first time to investigate probable changes in steroidogenesis and spermatogenesis of the adult DM-induced mice. Although some nonsignificant changes were noted in Dia-Ins-omg3 group compared with Dia-Ins group, the results were not encouraging. Other environmental factors affecting testicular functions such as X-ray irradiation, formaldehyde, and busulfan administration, as a model of spermatogenic dysfunction, might be utilized to understand probable impact of omega3 on damaged reproductive male organs.

## Figures and Tables

**Table 1:** Serum testosterone level and the number of Leydig cells were determined in the intact, diabetic-saline (Dia-Sa), diabetic-insulin (Dia-Ins), diabetic-omega3 (Dia-omg3), and diabetic-insulin-omega3 (Dia-Ins-omg3) groups, 35 days after the onset of DM. No statistically significant difference was noted among the groups.

	Intact	Dia-Sa	Dia-Ins	Dia-omg3	Dia-Ins-omg3
Leydig cells (*N*)	217.4 ± 34.4	229.3 ± 32.0	243 ± 19.5	255 ± 53.7	223.8 ± 36.7
Serum testosterone level (ng/ml)	1.94 ± 0.7	1.45 ± 0.5	1.26 ± 0.3	1.01 ± 0.2	1.44 ± 0.7

**Table 2:** Testis dimensions and volume were measured in the intact, diabetic-saline (Dia-Sa), diabetic-insulin (Dia-Ins), diabetic-omega3 (Dia-omg3), and diabetic-insulin-omega3 (Dia-Ins-omg3) groups by a standard caliper and digital balance. None of the parameters was statistically different among the groups.

	Intact	Dia-Sa	Dia-Ins	Dia-omg3	Dia-Ins-omg3
Testis volume (Cm^3^)	0.109 ± 0.008	0.095 ± 0.026	0.099 ± 0.012	0.100 ± 0.023	0.100 ± 0.017
Testis length (mm)	7.28 ± 0.31	6.60 ± 0.66	7.07 ± 0.32	6.98 ± 0.49	6.87 ± 0.54
Testis width (mm)	4.89 ± 0.27	4.65 ± 0.43	4.72 ± 0.24	4.49 ± 0.56	4.81 ± 0.94
Testis thickness (mm)	4.58 ± 0.35	4.40 ± 0.57	4.54 ± 0.95	4.61 ± 0.35	4.33 ± 0.27
